# Clinical Features of Idiopathic Interstitial Pneumonia with Systemic Sclerosis-Related Autoantibody in Comparison with Interstitial Pneumonia with Systemic Sclerosis

**DOI:** 10.1371/journal.pone.0161908

**Published:** 2016-08-26

**Authors:** Hideaki Yamakawa, Eri Hagiwara, Hideya Kitamura, Yumie Yamanaka, Satoshi Ikeda, Akimasa Sekine, Tomohisa Baba, Shinichiro Iso, Koji Okudela, Tae Iwasawa, Tamiko Takemura, Kazuyoshi Kuwano, Takashi Ogura

**Affiliations:** 1 Department of Respiratory Medicine, Kanagawa Cardiovascular and Respiratory Center, Yokohama, Japan; 2 Department of Radiology, Yokohama Rousai Hospital for Labour Welfare Corporation, Yokohama, Japan; 3 Department of Pathobiology, Yokohama City University Graduate School of Medicine, Yokohama, Japan; 4 Department of Radiology, Kanagawa Cardiovascular and Respiratory Center, Yokohama, Japan; 5 Department of Pathology, Japanese Red Cross Medical Center, Tokyo, Japan; 6 Department of Respiratory Medicine, Tokyo Jikei University Hospital, Tokyo, Japan; JAPAN

## Abstract

**Background:**

Patients with idiopathic interstitial pneumonias sometimes have a few features of connective tissue disease (CTD) and yet do not fulfil the diagnostic criteria for any specific CTD.

**Objective:**

This study was conducted to elucidate the characteristics, prognosis, and disease behavior in patients with interstitial lung disease (ILD) associated with systemic sclerosis (SSc)-related autoantibodies.

**Methods:**

We retrospectively analyzed medical records of 72 ILD patients: 40 patients with SSc (SSc-ILD) and 32 patients with SSc-related autoantibody-positive ILD but not with CTD (ScAb-ILD), indicating lung-dominant CTD with SSc-related autoantibody.

**Results:**

Patients with SSc-ILD were predominantly females and non-smokers, and most had nonspecific interstitial pneumonia confirmed by high-resolution computed tomography (HRCT) and pathological analysis. However, about half of the patients with ScAb-ILD were male and current or ex-smokers. On HRCT analysis, honeycombing was more predominant in patients with ScAb-ILD than with SSc-ILD. Pathological analysis showed the severity of vascular intimal or medial thickening in the SSc-ILD patients to be significantly higher than that in the ScAb-ILD patients. Survival curves showed that the patients with ScAb-ILD had a significantly poorer outcome than those with SSc-ILD.

**Conclusion:**

Data from this study suggest that lung-dominant CTD with SSc-related autoantibody is a different disease entity from SSc-ILD.

## Introduction

Connective tissue disease (CTD) is often associated with interstitial pneumonia. An evaluation for underlying CTD is recommended in the diagnosis and management of idiopathic interstitial pneumonias [1]. Some patients with idiopathic interstitial pneumonia have a few features of CTD and yet do not fulfil the diagnostic criteria for any specific CTD [2, 3]. These patients have been described previously as having undifferentiated CTD-associated interstitial lung disease (UCTD-ILD), lung-dominant CTD, and autoimmune-featured ILD, and the recently proposed term “interstitial pneumonia with autoimmune features” (IPAF) [1–8].

Among the various CTDs, rheumatoid arthritis (RA), systemic sclerosis (SSc), and polymyositis/dermatomyositis are more likely to be associated with ILD [9, 10]. Previous reports have indicated that clinical characteristics are similar between RA-ILD and ILD with anti-CCP antibody but not with RA, and between polymyositis/dermatomyositis-ILD and anti-ARS antibody-associated ILD but not with polymyositis/dermatomyositis [11, 12]. Specific study of each autoantibody found in ILD may be necessary to assess the appropriate strategy to diagnose and treat these disorders.

We hypothesized that ILD with SSc-related autoantibodies would not resemble SSc-ILD, and this point may contribute to the reasons for the difference prognoses in previous reports of UCTD-ILD, lung-dominant CTD, autoimmune-featured ILD, and IPAF [2–7, 13]. The aims of our study were thus to review and assess clinical characteristics, prognosis, and disease behavior of SSc-related autoantibody-associated ILD.

## Material and Methods

### Study sample

We retrospectively surveyed all patients who were diagnosed as having SSc-ILD or SSc-related autoantibody (anti-centromere, anti-scleroderma-70, and anti-U1 RNP antibody)-positive ILD but not CTD (ScAb-ILD) at Kanagawa Cardiovascular and Respiratory Center, Kanagawa, Japan, between March 1997 and July 2015. Among the patients with SSc-ILD, 3 patients with SSc-RA overlap and 1 patient with SSc- dermatomyositis overlap were excluded from this study. Moreover, among the patients with ScAb-ILD, 4 patients with RA, 4 patients with Sjögren’s syndrome, 2 patients with chronic hypersensitive pneumonitis, and 1 patient with IgG4-associated lung disease were also excluded. Diagnosis of SSc was made by rheumatologists at other institutions. Patients with SSc fulfilled the revised criteria for SSc of the American College of Rheumatology/European League Against Rheumatism (ACR/EULAR) classification [14]. In 4 patients with SSC-ILD, SSc preceded the onset of ILD (follow-up: 2.17–17.5 years). On the other hand, 2 patients developed manifestations of SSc during their follow-up period (5–7 months), and these patients were also included as SSc-ILD subjects. Although mixed connective tissue disease (MCTD) was originally described as a syndrome, the presence of anti-U1 RNP antibody is not restricted to MCTD but is also occasionally observed in patients with systemic lupus erythematosus and SSc [15]. Patients with SSc present with many disease features that are also found in MCTD, and if a single CTD becomes dominant, most patients who have MCTD develop SSc, and some MCTD patients often evolve toward severe SSc [16–18]. Therefore, in our study, 11 patients with MCTD were included as having SSc-ILD, and 15 anti-U1 RNP-positive patients with ILD were also included as having ScAb-ILD. Diagnosis of MCTD required fulfillment of at least one set of the three widely accepted diagnostic criteria (Sharp, Alarcon-Segovia, and Kasukawa criteria) [15, 19]. This study received approval from the institutional review board of Kanagawa Cardiovascular and Respiratory Center (no. 27–39). Informed consent was not required because of the retrospective nature of the study.

### Data collection

Baseline clinical measures, other than bronchoalveolar lavage and histological findings by surgical lung biopsy, were obtained within one month of the initial diagnosis of SSc-ILD or ScAb-ILD at our hospital. Bronchoalveolar lavage and histological findings by surgical lung biopsy were obtained within 3 months (27 patients), 3–6 months (4 patients), and 3.25 years (1 patient) of diagnosis. A broad panel of autoantibodies were screened in a clinical setting even if there were no symptoms suspicious of CTD. Anti-centromere, anti-scleroderma-70, and anti-U1 RNP antibodies were tested using fluoroenzymeimmunoassay testing of sera (Thermo Fischer Scientific Inc., Tokyo, Japan). A positive result for each autoantibody was defined as a measurement greater than 10 U/mL.

### Radiological analysis

Two radiologists (T. Iwasawa and S. Iso) reviewed high-resolution computed tomography (HRCT) scans for consensus of diagnosis of ILD in our hospital without information of each patient’s clinical data. Patients were classified as presenting a HRCT pattern either “suggestive or consistent with nonspecific interstitial pneumonia (NSIP)” or “suggestive of usual interstitial pneumonia (UIP)” [20, 21]. Pleuroparenchymal fibroelastosis was defined Reddy’s criteria [22]. The HRCT scans were analyzed for the following characteristics: honeycombing, ground-glass opacity, consolidation, reticulation, localization of attenuation in conjunction with honeycombing and cysts, traction bronchiectasis, bronchial wall thickening, pulmonary artery dilation, enlarged mediastinal lymph nodes, and pleural thickening. These features were selected on the basis of previous studies or from our experience [23–25]. Disagreements between the two radiologists after the first assessment were resolved by discussion. We also used computer-aided 3D quantitative analysis of chest HRCT to automatically categorize the lungs in the 3D CT images pixel-by-pixel with Gaussian histogram-normalized relations, and relative volume of disease extent (%) to HRCT lung volume was calculated [26, 27]. A previous study reported that an easily applicable, limited/extensive disease staging system for SSc-ILD based on combined evaluation with HRCT and pulmonary function testing (PFT) provides discriminatory prognostic information [28]. We thus used this staging system to categorize our study patients as having limited disease (HRCT extent ≤ 10% or, when HRCT extent was 10–30%, forced vital capacity [FVC] ≥ 70%) or extensive disease (HRCT > 30% or, when HRCT extent was 10–30%, FVC < 70%) [28].

### Pathological analysis

The surgical lung biopsy slides were reviewed by two pulmonary pathologists (K. Okudela and T. Takemura) who were blinded to the clinical and radiologic information. Histologic patterns were classified according to the current classification of idiopathic interstitial pneumonia [21]. Moreover, the following pathological features were semi-quantitatively graded as 0 (absent), 1 (mild), 2 (moderate), or 3 (severe): lung parenchyma, airway, and pleural lesions [29–31]. Any disagreements between the two pathologists were discussed until consensus was reached.

### Statistical analysis

Categorical baseline characteristics are summarized by frequency and percentage, and continuous characteristic are reported as mean ± SD. To detect differences between groups, the Wilcoxon test or Fisher’s exact test was used as appropriate. We investigated potential risk factors of mortality with each variable chosen for entry into univariate Cox regression analysis and then performed multivariate Cox regression analysis with backward variable selection. The Kaplan-Meier method was used to display and the log-rank test to compare survival curves for the cohort stratified for each group (SSc-ILD and ScAb-ILD; limited and extensive staging of disease; Krebs von den Lungen-6 (KL-6) ≥ 1000 U/mL and < 1000 U/mL; and anti-centromere, anti-scleroderma-70, and anti-U1RNP antibody). Analysis of disease behavior based on FVC over time was performed with linear mixed-effects models in which separate fits for subjects with SSc-ILD or ScAb-ILD were allowed. Each model included random terms for intercept and slope (for time from diagnosis) to account for the data structure (repeated measures over time within subject). We considered p < 0.05 to represent statistical significance in all analyses. Missing data were categorized as “unknown” and were entered into each statistical analysis model. All data were analyzed with SAS version 9.4 (SAS Institute Inc.).

## Results

### Patient characteristics

We identified 72 subjects of whom 40 were patients with SSc-ILD and 32 were patients with ScAb-ILD. Significantly more of the patients with SSc-ILD were women and never-smokers than were patients with ScAb-ILD (p = 0.013) (Table 1). The CD4/CD8 ratio of bronchoalveolar lavage fluid was significantly lower in the patients with SSc-ILD (p = 0.042). A quarter of the patients with SSc-ILD used cyclophosphamide. The median follow-up period was 2.51 years (range: 0.20–17.25 years) in SSc-ILD patients and 3.25 years (range: 0.15–9.75 years) in ScAb-ILD patients.

**Table 1:** Baseline characteristics at the time of diagnosis as interstitial lung disease.

Characteristics	All subjects	SSc-ILD	ScAb-ILD	*P* value
**No. of patients**	72	40	32	
**Female N (%)**	51 (70.8)	34 (85.0)	17 (53.1)	0.004*
**Age, mean ± SD**	65.3 ± 14.6	61.7 ± 16.4	69.7 ± 10.8	0.059
**Current or ex-smoker N (%)**	26 (36.1)	9 (22.5)	17 (53.1)	0.013*
**Body mass index, kg/m**^**2**^	22.18 ± 3.49	22.41 ± 3.18	21.89 ± 3.88	0.537
**Positive autoantibody N (%)**				
Anti-centromere antibody	26 (36.1)	13 (32.5)	13 (40.6)	0.161
Anti-scleroderma-70 antibody	15 (20.8)	11 (27.5)	4 (12.5)	
Anti-U1 RNP antibody	26 (36.1)	11 (27.5)	15 (46.9)	
**Lymphocyte, /μL**	1712.5 ± 644.2	1703.3 ± 601.8	1724.1 ± 703.3	0.747
**Hb, g/dL**	13.25 ± 1.44	13.14 ± 1.42	13.39 ± 1.47	0.289
**Albumin, g/dL**	3.96 ± 0.39	3.94 ± 0.38	3.98 ± 0.40	0.601
**LDH, IU/L**	248.0 ± 61.3	241.7 ± 64.4	255.9 ± 57.3	0.153
**CRP, mg/dL**	0.5 ± 0.892	0.462 ± 0.978	0.548 ±0.784	0.31
**KL-6 (available N)**	70	39	31	
U/mL	1307.4 ± 951.2	1266.9 ± 938.0	1358.4 ± 980.6	0.727
≥ 1000 U/mL N (%)	36 (50.0)	19 (47.5)	17 (53.1)	0.638
**SP-D (available N)**	64	36	28	
ng/mL	190.52 ± 130.65	196.61 ± 141.94	182.7 ± 116.61	0.855
≥ 110 ng/mL N (%)	43 (59.7)	24 (60.0)	19 (59.4)	1
**Pulmonary function tests**				
Subjects (available N)	65	39	26	
FEV_1_/ FVC ratio, %	79.8 ± 10.7	80.1 ± 8.6	79.4 ± 13.4	0.952
FVC, % predicted	85.74 ± 21.04	83.97 ± 20.61	88.39 ± 21.81	0.547
≥ 70% N	50	29	21	0.765
Subjects (available N)	55	34	21	
D_LCO_, % predicted	70.0 ± 20.94	69.41 ± 19.44	70.94 ± 23.65	1
≥ 50% N	46	29	17	0.719
**Staging**				
Limited disease N (%)	33 (45.8)	19 (47.5)	14 (43.8)	1
Extensive disease N (%)	33 (45.8)	18 (45.0)	15 (46.9)	
Unknown N (%)	6 (8.3)	3 (7.5)	3 (9.4)	
**Bronchoalveolar lavage**				
Subjects (available N)	25	18	7	
Total cells (×10^4^ mL)	27.4 ± 19.08	27.45 ± 19.26	27.28 ± 20.39	0.685
CD4/ CD8 ratio	1.393 ± 1.422	1.141 ± 1.230	2.065 ± 1.790	0.042*
Lymphocytes, %	19.91 ± 13.74	17.69 ± 14.76	25.60 ± 9.22	0.115
Neutrophils, %	6.666 ± 6.470	7.319 ± 7.265	4.986 ± 3.654	0.785
Eosinophils, %	3.770 ± 4.710	4.340 ± 5.300	2.290 ± 2.360	0.543
**Medication (during follow-up), N (%)**				
PAH-specific drug therapy use†	4 (5.6)	4 (10.0)	0 (0.0)	0.124
Steroid use	30 (41.7)	19 (47.5)	11 (34.4)	0.338
Cyclophosphamid use	6 (8.3)	6 (15.0)	0 (0.0)	0.03*
Cyclosporine or tacrolimus or azathioprine use	15 (20.8)	9 (22.5)	6 (18.8)	0.776
Pirfenidone use	5 (6.9)	1 (2.5)	4 (12.5)	0.164
**Deaths N (%)**	18 (25.0)	5 (12.5)	13 (40.6)	
Cause of death N				
Interstitial pneumonia	10	1	9	
Bacterial pneumonia	2	2	0	
Cardiac or cerebral infarction	3	0	3	
Breast cancer	1	1	0	
Unknown	2	1	1	
**Median follow-up years (range)**	2.84 (0.15–17.25)	2.51 (0.20–17.25)	3.25 (0.15–9.75)	

Data are presented as mean ± SD, unless otherwise stated. SSc: systemic sclerosis; ILD: interstitial lung disease; Ab: autoantibody; Hb: hemoglobin; LDH: lactate dehydrogenase; CRP: C-reactive protein; SP-D: surfactant protein-D; FEV_1_: forced expiratory volume in 1 s; FVC: forced vital capacity; D_LCO_: diffusing capacity of the lung for carbon monoxide; PAH: pulmonary arterial hypertension.

^†^PAH-specific drugs include beraprost sodium (N = 2) and sildenafil (N = 2).

*P value less than 0.05.

### Radiographic features

**Fig 1:**
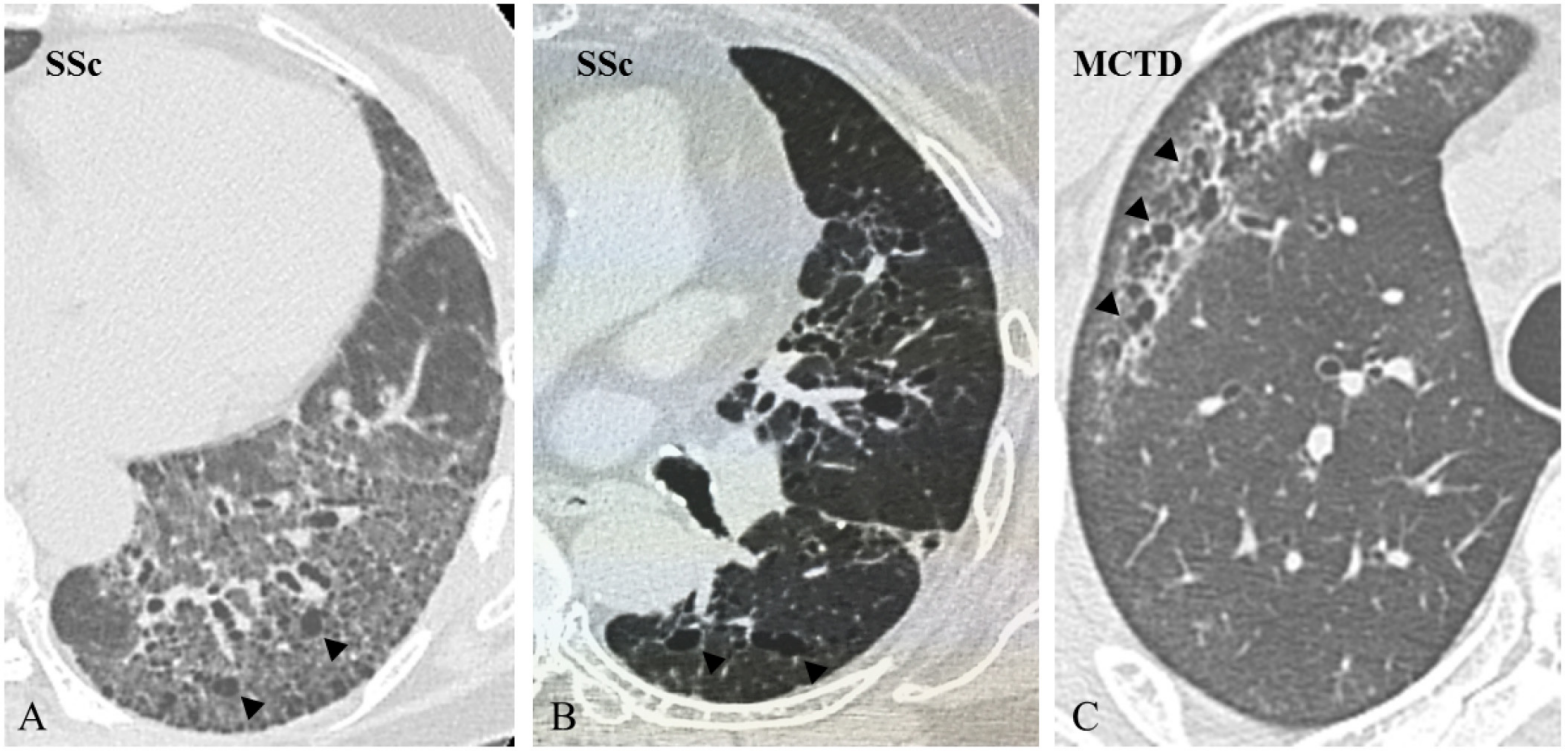
HRCT scans of cyst formation. (A-B) HRCT scan demonstrates cyst formation (arrowheads) with pulmonary fibrosis, traction bronchiectasis, and architectural distortion in two patients with SSc-ILD. There is no continuity between the cysts and traction bronchiectasis. (C) HRCT scan shows cysts (arrowheads) with opacity separated from the pleura in a patient with MCTD-ILD.

Radiographic features in SSc-ILD included UIP in 3 (7.5%) patients and NSIP in 32 (80.0%), and in ScAb-ILD included UIP in 7 (21.9%) patients and NSIP in 16 (50.0%), indicating that NSIP was a more frequent pattern in SSc-ILD, and the frequency of UIP was slightly more common in ScAb-ILD than in SSc-ILD (p = 0.024) (Table 2). Honeycombing was observed significantly more frequently in ScAb-ILD (p = 0.003). In contrast, cystic changes (non-honeycombing, emphysema) were significantly more frequent in SSc-ILD (p = 0.013) (Fig 1).

**Table 2:** Comparison of HRCT and pathological findings between SSc-ILD and ScAb-ILD.

Characteristics	All subjects	SSc-ILD	ScAb-ILD	*P* value
**No. of patients**	72	40	32	
**HRCT pattern N (%)**				
Suggestive of UIP	10 (13.9)	3 (7.5)	7 (21.9)	0.024†*
Suggestive or consistent with NSIP	48 (66.7)	32 (80.0)	16 (50.0)	
Others††	14 (19.4)	5 (12.5)	9 (28.1)	
**HRCT findings N (%)**				
GGO	65 (90.3)	38 (95.0)	27 (84.4)	0.23
Distribution (unilateral/ bilateral)	2 (2.8)/ 63 (87.5)	1 (2.5)/ 37 (92.5)	1 (3.1)/ 26 (81.3)	1
(upper/ lower/ diffuse or random)	1 (1.4)/ 49 (68.1)/ 15 (20.8)	0 (0.0)/ 32 (80.0)/ 6 (15.0)	1 (3.1)/ 17 (53.1)/ 9 (28.1)	0.097
(peribronchovascular/ subpleural/ diffuse)	14 (19.4)/ 21 (29.2)/ 30 (41.7)	7 (17.5)/ 15 (37.5)/ 16 (40.0)	7 (21.9)/ 6 (18.8)/ 14 (43.8)	0.333
Consolidation	17 (23.6)	8 (20.0)	9 (28.1)	0.577
Distribution (unilateral/ bilateral)	3 (4.2)/ 14 (19.4)	2 (5.0)/ 6 (15.0)	1 (3.1)/ 8 (25.0)	0.576
(upper/ lower/ diffuse or random)	4 (5.6)/ 12 (16.7)/ 1 (1.4)	1 (2.5)/ 6 (15.0)/ 1 (2.5)	3 (9.4)/ 6 (18.8)/ 0 (0.0)	0.576
(peribronchovascular/ subpleural/ diffuse)	2 (2.8)/ 8 (11.1)/ 7 (9.7)	2 (5.0)/ 1 (2.5)/ 5 (12.5)	0 (0.0)/ 7 (21.9)/ 2 (6.3)	0.016*
Reticulation	68 (94.4)	39 (97.5)	29 (90.6)	0.317
Distribution (unilateral/ bilateral)	3 (4.2)/ 65 (90.3)	1 (2.5)/ 39 (97.5)	2 (6.3)/ 27 (84.4)	0.571
(upper/ lower/ diffuse or random)	3 (4.2)/ 62 (86.1)/ 3 (4.2)	1 (2.5)/ 35 (87.5)/ 3 (7.5)	2 (6.3)/ 27 (84.4)/ 0 (0.0)	0.293
(peribronchovascular/ subpleural/ diffuse)	16 (22.2)/ 32 (44.4)/ 20 (27.8)	11 (27.5)/ 16 (40.0)/ 12 (30.0)	5 (15.6)/ 16 (50.0)/ 8 (25.0)	0.51
Honeycombing	15 (20.8)	3 (7.5)	12 (37.5)	0.003*
Traction bronchiectasis	61 (84.7)	32 (80.0)	29 (90.6)	0.325
Bronchial wall thickening	57 (79.2)	29 (72.5)	28 (87.5)	0.151
Micro-nodules	10 (13.9)	5 (12.5)	5 (15.6)	0.743
Emphysema	18 (25.0)	8 (20.0)	10 (31.3)	0.29
Cyst (non-honeycombing, emphysema)	17 (23.6)	14 (35.0)	3 (9.4)	0.013*
Mosaic attenuation (air trapping)	32 (44.4)	17 (42.5)	15 (46.9)	0.813
Enlarged mediastinal lymph node	14 (19.4)	8 (20.0)	6 (18.8)	1
Pleural thickening or effusion	7 (9.7)	4 (10.0)	3 (9.4)	1
Pulmonary artery dilatation	27 (37.5)	19 (47.5)	8 (25.0)	0.056
Volume loss	56 (77.8)	32 (80.0)	24 (75.0)	0.776
**HRCT**				
Disease extent, (available N)	69	38	31	
%	30.79 ± 15.327	28.638 ± 12.676	33.429 ± 17.923	0.379
≥ 30% N (%)	32 (44.4)	17 (42.5)	15 (46.9)	0.812
**Pathological pattern (available N)**	32	25	7	
UIP	1 (1.4)	1 (2.5)	0 (0.0)	0.085†
Fibrotic NSIP	20 (27.8)	18 (45.0)	2 (6.3)	
Unclassifiable	11 (15.3)	6 (15.0)	5 (15.6)	
**Pathological features, grade 0/ 1/ 2/ 3**				
Lung parenchyma lesion				
Cellular infiltration	0/ 10/ 16/ 6	0/ 8/ 11/ 6	0/ 2/ 5/ 0	0.085
Plasma cell infiltration	0/ 10/ 13/ 9	0/ 8/ 9/ 8	0/ 2/ 4/1	0.698
Lymphoid follicle with germinal center	15/ 14/ 2/ 1	10/ 12/ 2/ 1	5/ 2/ 0/ 0	0.13
Fibrosis	0/ 4/ 17/ 11	0/ 3/ 13/ 9	0/ 1/ 4/ 2	0.742
Honeycombing	24/ 7/ 1/ 0	19/ 5/ 1/ 0	5/ 2/ 0/ 0	0.88
Fibroblastic foci	10/ 19/ 1/ 2	7/ 16/ 1/ 1	3/ 3/ 0/ 1	0.714
Organizing pneumonia (intra-alveolar polyp)	16/ 12/ 3/ 1	15/ 8/ 1/ 1	1/ 4/ 2/ 0	0.033*
Atelectasis (collapse)	3/ 12/ 9/ 8	3/ 9/ 6/ 7	0/ 3/ 3/ 1	0.962
Cyst formation	25/ 4/ 3/ 0	20/ 3/ 2/ 0	5/ 1/ 1/ 0	0.636
Airway lesion				
Cellular infiltration	2/ 20/ 5/ 5	2/ 14/ 4/ 5	0/ 6/ 1/ 0	0.429
Lymphoid follicle	26/ 5/ 1/ 0	21/ 3/ 1/ 0	5/ 2/ 0/ 0	0.523
Fibrosis	29/ 3/ 0/ 0	22/ 3/ 0/ 0	7/ 0/ 0/ 0	1
Traction bronchiectasis	9/ 16/ 6/ 1	7/ 11/ 6/ 1	2/ 5/ 0/ 0	0.386
Vascular intimal or medial thickening	19/ 8/ 4/ 1	12/ 8/ 4/ 1	7/ 0/ 0/ 0	0.020*
Pleural fibrosis	8/ 21/ 3/ 0	6/ 18/ 1/ 0	2/ 3/ 2/ 0	0.514
Pleural inflammation	17/ 10/ 4/ 1	12/ 8/ 4/ 1	5/ 2/ 0/ 0	0.208
Smoking-related lesion				
Emphysema	13/ 13/ 6/ 0	11/ 10/ 4/ 0	2/ 3/ 2/ 0	0.403
Respiratory bronchiolitis	27/ 4/ 1/ 0	23/ 2/ 0/ 0	4/ 2/ 1/ 0	0.025*
Bronchial metaplasia	9/ 14/ 7/ 2	8/ 9/ 6/ 2	1/ 5/ 1/ 0	0.981
DIP reaction	17/ 10/ 5/ 0	14/ 8/ 3/ 0	3/ 2/ 2/ 0	0.42

Data are presented as mean ± SD, unless otherwise stated. SSc: systemic sclerosis; ILD: interstitial lung disease; Ab: autoantibody; HRCT: high-resolution computed tomography; UIP: usual interstitial pneumonia; NSIP: nonspecific interstitial pneumonia; GGO: ground glass opacity; DIP: desquamative interstitial pneumonia. †In relation to HRCT pattern except for others.

^††^Others includes cases with pleuroparenchymal fibroelastosis (N = 4) and unclassifiable (N = 10).

^†^*P* values were calculated except others.

*P value less than 0.05

### Pathologic features

**Fig 2:**
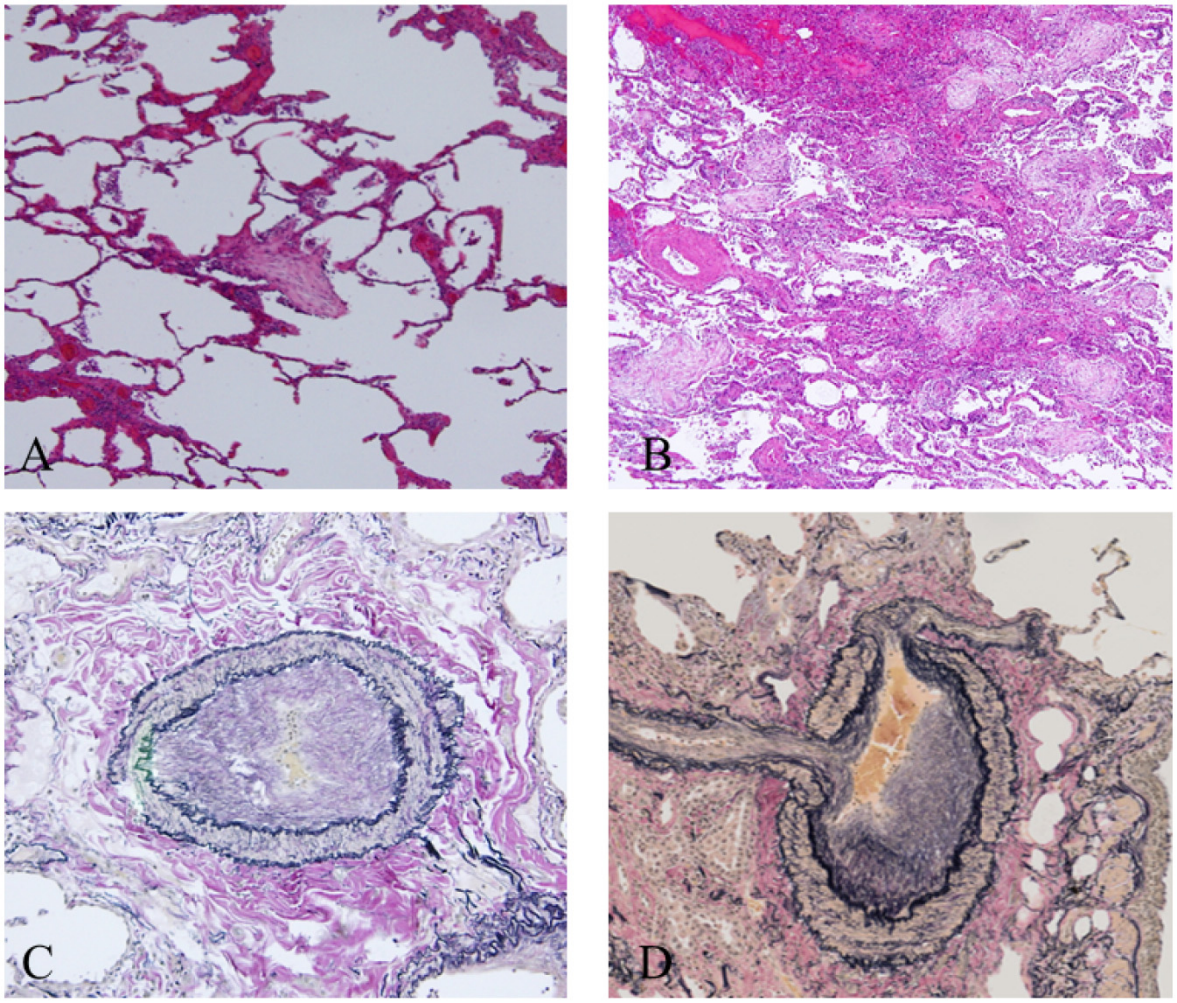
Examples of pathological scoring. (A-B) Typical imaging in each grade of organizing pneumonia (intra-alveolar polyps) as found in patients with ScAb-ILD (hematoxylin-eosin stain) ([A]: grade 1 and [B]: grade 3). (C-D) Vascular intimal or medial thickening as found in patients SSc-ILD (Elastica van Gieson stain) ([C]: grade 1 and [D] grade 3].

Of the 72 patients, 32 underwent surgical lung biopsy for diagnosis of ILD. Of the 25 patients with SSc-ILD, the major histologic pattern was fibrotic NSIP in 18 patients, UIP in 1 patient, and “unclassifiable” in 6 patients (Table 2). The histologic pattern in the 7 patients with ScAb-ILD included “unclassifiable” in 5 patients, fibrotic NSIP in 2 patients, and UIP in none. Organizing pneumonia (intra-alveolar polyps) and respiratory bronchiolitis were present in 40% and 8% of the patients with SSc-ILD and in 85.7% and 42.9%, respectively, of the patients with ScAb-ILD, indicating significantly more frequent findings of organizing pneumonia in ScAb-ILD (p = 0.033). The severity of vascular intimal or medial thickening in the patients with SSc-ILD was significantly higher than that in those with ScAb-ILD (p = 0.020). Typical examples of pathological features in each grade are shown in Fig 2.

### Mortality

Death from any cause occurred in 18 patients (20.0%) over a median 2.84-year follow-up period (range: 0.15–17.25 years). The overall cumulative 5-year mortality rate was 24.4%, whereas that of the patients with SSc-ILD and with ScAb-ILD was 10.9% and 35.9%, respectively (Table 1).

### Prognostic factors of all-cause mortality

**Fig 3:**
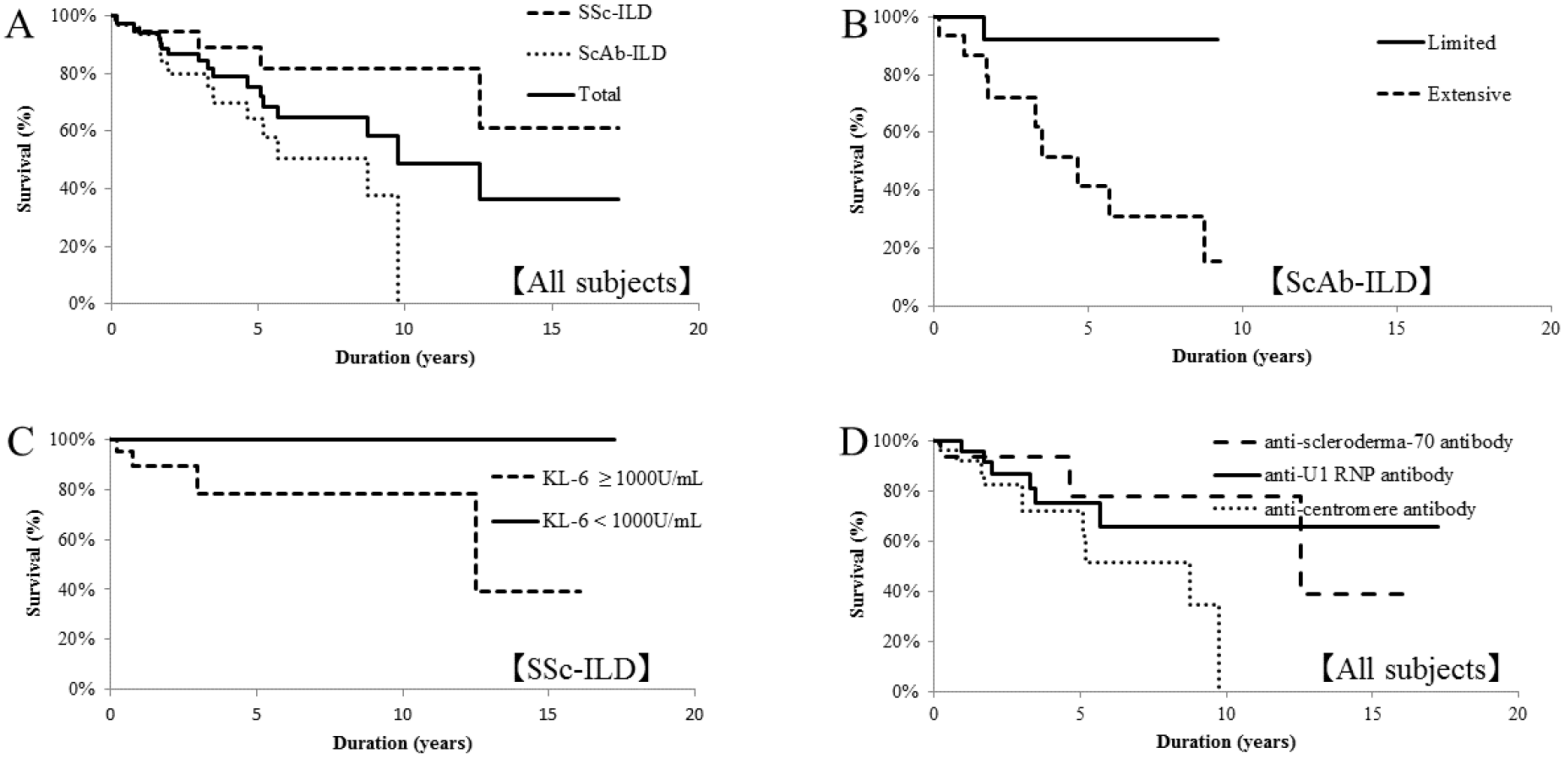
Kaplan-Meier survival curves of all-cause mortality. Overall cumulative 5-year mortality was 24.4%. (A) Patients with ScAb-ILD (dotted line) had worse survival than those with SSc-ILD (dashed line) (p = 0.011). (B) In patients with ScAb-ILD, those with extensive disease (dashed line) had worse survival than those with limited disease (solid line) (p = 0.015). (C) In patients with SSc-ILD, those with KL-6 ≥ 1000 U/mL (dashed line) had worse survival than those with KL-6 < 1000 U/mL (solid line) (p = 0.049). (D) The survival curves for patients with each type of autoantibody were not significantly different (p = 0.905 for comparison between anti-scleroderma-70 and anti-U1 RNP antibody, p = 0.089 for comparison between anti-scleroderma-70 and anti-centromere antibody, and p = 0.137 for comparison between anti-U1 RNP and anti-centromere antibody).

Log-rank testing showed that subjects with ScAb-ILD had significantly worse survival than those with SSc-ILD (p = 0.011) (Fig 3A). Analysis of staging (limited *versus* extensive disease) of all subjects and those with SSc-ILD showed no significant differences (p = 0.120 and 0.338, respectively), whereas just for ScAb-ILD, the patients with extensive disease had worse survival than those with limited disease (p = 0.015) (Fig 3B). For serum KL-6 in just the SSc-ILD patients, those with KL-6 ≥ 1000 U/mL had worse survival than those with KL-6 < 1000 U/mL (p = 0.049) (Fig 3C). In the analysis of each autoantibody, no significant difference in survival was found between patients with anti-centromere, anti-scleroderma-70, and anti-U1 RNP antibody (Fig 3D). ScAb-ILD, age ≥ 65 years, CRP ≥ 1 mg/dL, and honeycombing on HRCT were significant predictors of mortality in univariate analysis. A multivariate Cox proportional hazard model showed only age ≥ 65 years and CRP ≥ 1 mg/dL to be negative prognostic factors (Table 3).

**Table 3:** Analysis of factors associated with mortality.

	Univariate Cox Regression		Multivariate Cox Regression	
Characteristics	Crude HR (95% CI)	*P* value	Adjusted HR (95% CI)	*P* value
**Disease type**				
ScAb-ILD	3.973 (1.267–12.464)	0.018*	1.189 (0.337–4.199)	0.788
**Autoantibody**				
Anti-scleroderma-70 antibody	Reference			
Anti-centromere antibody	2.649 (0.682–10.284)	0.159		
Anti-U1 RNP antibody	1.142 (0.283–4.607)	0.852		
**Age, ≥ 65 years**	8.067 (1.824–35.687)	0.006*	8.794 (1.878–41.186)	0.006*
**Ever-smoker**	1.312 (0.512–3.357)	0.572		
**FVC, < 70%**	1.115 (0.338–3.682)	0.858		
**DLCO, < 50%**	1.277 (0.268–6.088)	0.759		
**CRP, ≥ 1 mg/dL**	5.070 (1.874–13.717)	0.001*	7.917 (2.303–27.211)	0.001*
**KL-6, ≥ 1000 U/mL**	1.031 (0.383–2.776)	0.952		
**Disease extent on HRCT, ≥ 30%**	1.599 (0.608–4.205)	0.342		
**Extensive disease**	2.410 (0.755–7.692)	0.137		
**UIP pattern on HRCT**	1.017 (0.214–4.519)	0.983		
**Honeycombing on HRCT**	4.301 (1.582–11.691)	0.004*	0.482 (0.087–2.651)	0.401

Ab: autoantibody; ILD: interstitial lung disease; CRP: C-reactive protein; FVC: forced vital capacity; D_LCO_: diffusing capacity of the lung for carbon monoxide; HRCT: high-resolution computed tomography; UIP: usual interstitial pneumonia.

*P value less than 0.05.

### Disease behavior during follow-up

**Fig 4:**
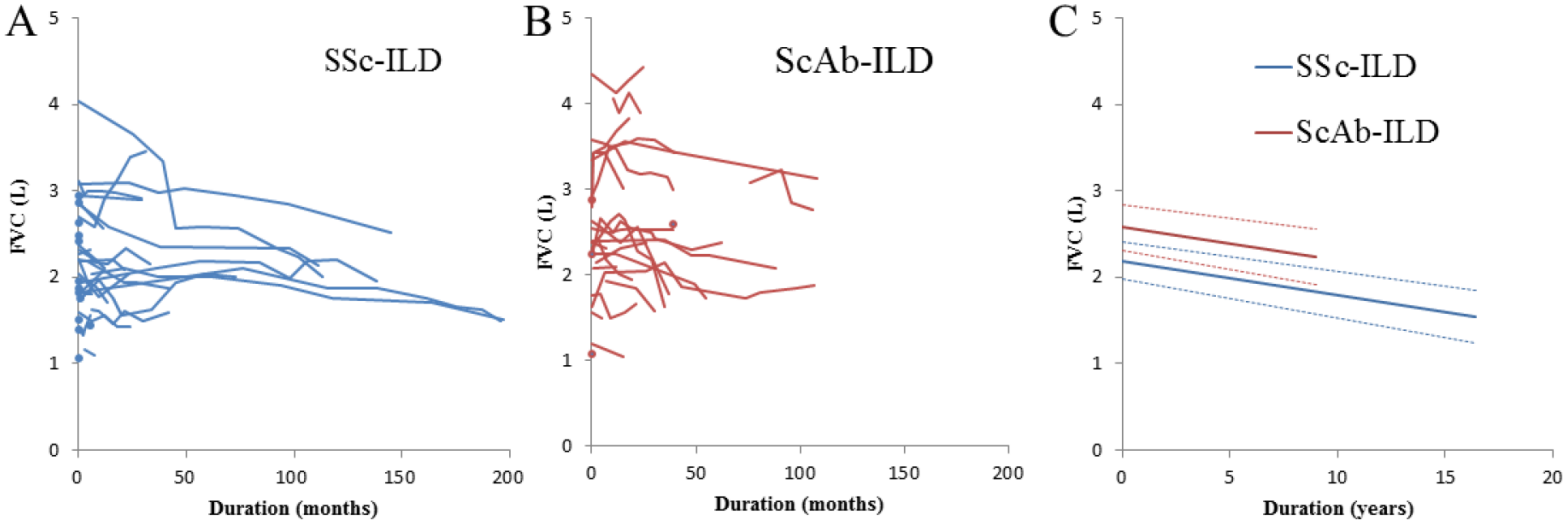
Changes in forced vital capacity (FVC) during follow-up. (A) SSc-ILD (B) ScAb-ILD (C) Regression lines were calculated by solving the linear mixed-effects model. Baseline FVC (intercept) with ScAb-ILD (mean, 2.574 L [95% CI: 2.313–2.835]) was significantly higher than that with SSc-ILD (mean, 2.191 L [95% CI: 1.974–2.407]) (p = 0.027). The declining slopes of FVC between both groups were not significantly different (SSc-ILD: mean, -0.03979 L year^-1^ [95% CI: -0.05449 to -0.02509]; ScAb-ILD: mean, -0.03740 L year^-1^ [95% CI: -0.06446 to -0.01034] (p = 0.878).

Baseline FVC (intercept) in ScAb-ILD (mean, 2.574 L [95% confidence interval (CI): 2.313–2.835]) was significantly higher than that in SSc-ILD (mean, 2.191 L [95% CI: 1.974–2.407]) (p = 0.027). The declining slopes of FVC were not significantly different between the two groups (SSc-ILD: mean, -0.03979 L year^-1^ [95% CI: -0.05449 to -0.02509]; ScAb-ILD: mean, -0.03740 L year^-1^ [95% CI: -0.06446 to -0.01034] (p = 0.878) (Fig 4).

## Discussion

The present study clarified the differences in patient characteristics, disease behavior, and prognosis between SSc-ILD and ScAb-ILD and showed that lung-dominant CTD with ScAb-ILD is a different disease entity from that of SSc-ILD. Although analyses of SSc-ILD were often reported previously, to the best of our knowledge, the association between SSc-ILD and SSc-related autoantibody-positive ILD but not SSc indicates that lung-dominant CTD has not been reported before.

SSc is a CTD characterized by tissue fibrosis in the skin and internal organs. ILD develops in more than half of the patients with SSc and is an important risk factor of mortality as is pulmonary hypertension [32, 33]. As in previous studies, our patients with SSc-ILD were more likely to be females and non-smokers, and most cases were diagnosed as NSIP through both HRCT and pathological analysis [34]. In contrast, about half of the patients with ScAb-ILD were male and current or ex-smokers.

In HRCT analysis, although one-half of the patients with ScAb-ILD were diagnosed as having NSIP, over one-fifth of them had UIP, and honeycombing was more predominant in those with ScAb-ILD rather than SSc-ILD. Interestingly, HRCT findings of cysts were more likely to be present in those with SSc-ILD rather than ScAb-ILD. Certain segments of the population have secondary Sjögren's syndrome associated with SSc, and thin-walled cysts are often seen in Sjögren's syndrome with ILD [35]. In our study, two patients were diagnosed as having secondary Sjögren's syndrome with SSc. The precise frequency of such patients may actually be higher in our study and thus might lead to significantly different results.

Pathological analysis showed the severity of vascular intimal or medial thickening to be significantly higher in the patients with SSc-ILD than ScAb-ILD. Although only a few patients underwent specific drug therapy for pulmonary arterial hypertension in our study, the primary findings in SSc patients are intimal fibrosis (affecting the small vessels adjacent to the alveoli), medial hyperplasia, and adventitial fibrosis affecting the pulmonary arterioles; thus, our results were compatible with these findings [36]. In contrast, none of the patients with ScAb-ILD had such lesions. Moreover, the histologic pattern of most patients (71.4%) with ScAb-ILD who underwent surgical lung biopsy was “unclassifiable.” The high frequency of smoking history in the ScAb-ILD patients and the existence of the autoantibodies themselves may contribute to this result, but the exact cause remains unclear.

The survival curves showed that patients with ScAb-ILD had a significantly poorer outcome than those with SSc-ILD. Although the ScAb-ILD patients with extensive disease had a significantly poorer survival curve than those with limited disease, there was no significant difference in prognosis between the SSc-ILD patients with limited *versus* extensive disease. A previous study reported that the limited/extensive staging system strongly predicted mortality [28]. The SSc-ILD patients in our study did not show the same result, and the small sample size might be one reason for this finding. The SSc-ILD patients, and not the ScAb-ILD patients, with the biomarker of serum KL-6 ≥ 1000 U/mL had a worse survival curve than those with KL-6 < 1000 U/mL. A serum KL-6 of ≥ 1000 U/mL was previously reported to be a predictor of poor prognosis in patients with idiopathic pulmonary fibrosis [37], and in SSc-ILD, the presence of elevated KL-6 values is also a poor prognostic factor [38].

When disease behavior was assessed on the basis of FVC, the declining slope of FVC was not significantly different between the two groups. SSc-ILD was reported to progress much more frequently in the first 4 years of systemic disease (especially in the first 2 years) [39], but similar results were not seen in our study. Interestingly, despite the severity of vascular intimal and medial thickening in the pathological findings of the patients with SSc-ILD and the baseline FVC calculated by solving the linear mixed-effects model being significantly higher in those with ScAb-ILD, the patients with ScAb-ILD appeared to have a worse prognosis. Disease behavior related to the declining slope of FVC was not significantly different between the two groups, which indicates that there was missing data during follow-up period because of the poor prognosis of the patients with ScAb-ILD.

To summarize, there were many differences between the baseline characteristics and HRCT and pathological findings of the two disease entities, and primarily, the prognosis of the patients with ScAb-ILD was poorer than that of the patients with SSc-ILD. All of our subjects fulfilled the criteria for lung-dominant CTD proposed by Fischer et al. [1]. Most of the subjects with ScAb-ILD matched the diagnostic criteria for IPAF because these patients have a serologic domain (anti-centromere, anti-scleroderma-70, and anti-U1 RNP antibodies) and morphologic domain as indicated predominantly by radiological findings of NSIP, intrinsic airway disease with bronchial wall thickening and air trapping, and pathological findings, and by cellular infiltration of the airway and organizing pneumonia. Therefore, in our investigation of these many aspects, we suggest that patients with ILD who have not met the diagnosis of SSc but are positive for SSc-related autoantibodies have a disease entity distinct from that of SSc-ILD, and this is an important reason for the difference in some reports between the prognosis of CTD-ILD *versus* that of UCTD-ILD, lung-dominant CTD, autoimmune-featured ILD, and IPAF.

The limitations of this study are as follows. First, the study involved a relatively small number of patients from a single center. However, in our institution, chest clinicians carry out screening while always keeping routine examination of each CTD-related autoantibody in mind, even if the patients with ILD have no symptoms suspicious of CTD. Moreover, if the patients have autoantibodies related to CTD, we usually consult the rheumatologists to determine whether the diagnosis of CTD can be fulfilled. Therefore, we could examine the patients in greater detail. Second, the patients with MCTD were included as patients with SSc in this study. As mentioned in the *Study sample* section, anti-U1 RNP antibody is occasionally observed in patients with SSc and systemic lupus erythematosus, and chronic interstitial pneumonitis as a complication of systemic lupus erythematosus is rare [14, 40]. Moreover, most patients with MCTD have characteristics of SSc and ultimately develop SSc [16–18]. In fact, 7 (63.6%) of the 11 patients with MCTD fulfilled the criteria for SSc during the follow-up period. Therefore, ILD with MCTD is included in SSc-ILD, and ILD with positive U1 RNP antibody is included in ScAb-ILD.

We conclude that despite these limitations, our study suggests that lung-dominant CTD and IPAF with SSc-related autoantibody is a different disease entity from that of SSc-ILD. We believe that the results of our study will be helpful in determining whether the management of IPAF should be similar to that of CTD-ILD. Further studies with each of the specific autoantibodies of IPAF may be necessary to assess the appropriate strategy to diagnose and treat IPAF.
